# Combined fine-needle aspiration and selective intraoperative frozen section to optimize prediction of malignant thyroid nodules: A retrospective cohort study of more than 3000 patients

**DOI:** 10.3389/fendo.2023.1091200

**Published:** 2023-02-06

**Authors:** Zhuochao Mao, Yongfeng Ding, Liping Wen, Yu Zhang, Guofa Wu, Qihan You, Jie Wu, Dingcun Luo, Lisong Teng, Weibin Wang

**Affiliations:** ^1^ Department of Surgical Oncology, The First Affiliated Hospital, Zhejiang University School of Medicine, Hangzhou, Zhejiang, China; ^2^ Key Laboratory of Precision Diagnosis and Treatment for Hepatobiliary and Pancreatic Tumor of Zhejiang Province, First Affiliated Hospital, School of Medicine, Zhejiang University, Hangzhou, China; ^3^ Department of Medical Oncology, The First Affiliated Hospital, Zhejiang University School of Medicine, Hangzhou, China; ^4^ Department of Surgical Oncology, Affiliated Hangzhou First People's Hospital, Zhejiang University School of Medicine, Hangzhou, Zhejiang, China; ^5^ Department of General Surgery (Thyroid and Breast Surgery), The People’s Hospital of Haining City, Haining, China; ^6^ Department of Pathology, Affiliated Hangzhou First People’s Hospital, Zhejiang University School of Medicine, Hangzhou, China; ^7^ State Key Laboratory for Diagnosis and Treatment of Infectious Diseases, National Clinical Research Center for Infectious Diseases, Collaborative Innovation Center for Diagnosis and Treatment of Infectious Diseases, The First Affiliated Hospital, College of Medicine, Zhejiang University, Hangzhou, China

**Keywords:** FNA, FS, thyroid nodule, prediction, diagnostic model

## Abstract

**Background:**

Preoperative fine-needle aspiration (FNA) is widely used to differentiate malignant from benign thyroid nodules, while intraoperative frozen sections (FS) are suggested as a systematic supplement for intraoperative decision-making, but limitations still remain for both procedures.

**Methods:**

Medical records of 3807 patients with thyroid nodules who underwent both pathological diagnoses (FS and FNA) at our hospital were reviewed. The diagnostic accuracy, sensitivity, specificity, positive predictive value (PPV), and negative predictive value (NPV) of FNA and FS were also evaluated. We further designed an optimal integration scheme (FNA+selective FS) to predict thyroid nodule malignancy. Finally, the efficiency of the proposed integrated diagnostic model was validated using an independent external cohort.

**Results:**

For distinguishing malignant nodules, FNA had an accuracy of 90.3%, sensitivity of 90.7%, specificity of 85.2%, PPV of 98.8% and NPV of 40.4%. In contrast, the FS represented higher discriminative power (Accuracy, 94.5%; Sensitivity, 94.1%; Specificity, 100%; PPV, 100%; and NPV, 55.6%). we proposed the selective usage of FS (removed nodules with Bethesda category VI from routine FS, ~1/3 of total). The integrated new diagnostic model of FNA plus selective FS (FNA+sFS) achieved accuracy of 96.9%, sensitivity of 97.3%, specificity of 92%, PPV of 99.4%, and NPV of 71.6% (NRI=0.135, 95% CI 0.103-0.167, P <0.001) and was successfully applied to an external cohort (N=554).

**Conclusion:**

Compared with the FNA diagnostic system, FS has an increased ability to distinguish benign and malignant thyroid nodules. The newly proposed integrated diagnostic model of FNA + selective FS can optimize the accuracy of diagnosis.

## Introduction

The incidence of thyroid cancer, the most common endocrine malignancy, has been on an upward trend worldwide in recent decades (1, 2). New cases in China are estimated to be 90,000 per year and increasing significantly (3, 4). With the extensive use of high-resolution B-ultrasound in recent years, thyroid nodules can easily be detected in up to 60%-70% of the population, especially in Asian countries (5, 6). Because most of these nodules are benign and require no further treatment, researchers and clinicians have invested much effort in optimizing the filtering process to rule out malignant nodules.

The accurate screening of malignant nodules has long been a major challenge in the management of thyroid disease. For suspicious nodules detected by B-ultrasound, guidelines in every country differ in diagnostic methods such as fine-needle aspiration (FNA) and frozen sections (FS) (7–9). FNA is the most widely used procedure for cytology, which is defined by the Bethesda system depending on the probability of malignancy (10).

FNA can distinguish approximately 75%-80% of nodules, while the remaining 20%-25% remain indeterminate. Some other supplementary molecular tools can help “rule out” (Afirma GSC) or “rule in” (Thyroseq v3) malignancy in such cases, however, it can only validate 50%-60% of these indeterminate nodules and is unavailable in many countries (11, 12). Intraoperative FS is another widely performed diagnostic procedure which provides a quick intraoperative assessment of nuclear features of thyroid nodules (9). It is routinely used in a lot of countries including China, but the real clinical value and necessity of FS is still highly debated, especially in high Bethesda categories such as Bethesda V and VI (13, 14).

To evaluate the clinical values of fine needle aspiration and intraoperative frozen sections, we retrospectively analyzed 3807 patients who underwent thyroidectomy at our hospital with complete pathological information of FNA, FS, and final pathology (FP) from 2012 to 2016. With the validation of each diagnostic procedure, we propose a modified prediction pathway of “FNA+ selective FS” to optimize predictive accuracy and medical cost efficiency.

## Materials and methods

### Patients

**Figure 1:**
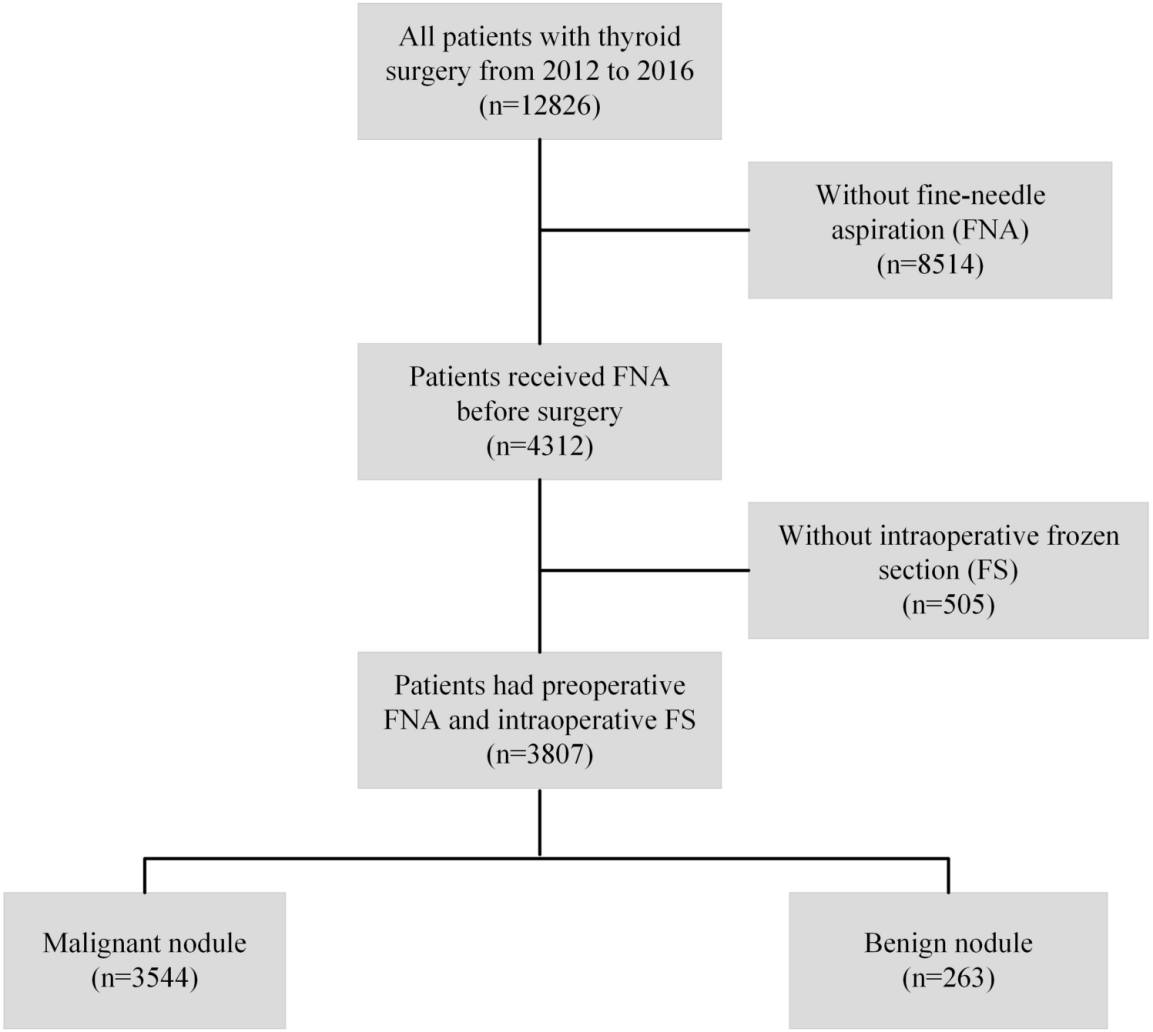
Study cohort flowchart.

This study included patients who underwent thyroid surgery between January 2012 and December 2016 at the First Affiliated Hospital of Zhejiang University School of Medicine. To explore the pathological consistency among FNA, FS, and FP of thyroid nodules, inclusion and exclusion criteria were defined and are listed in **Figure 1**. Patients who did not undergo FNA or FS were excluded from the study. Similarly, an independent cohort of patients who underwent thyroid surgery at Hangzhou First People’s Hospital, Zhejiang Province, China (from May 2015 to March 2020) was included in this study according to the procedures described above. This study was approved by the Institutional Review Board of the First Affiliated Hospital of Zhejiang University School of Medicine and Hangzhou First People’s Hospital. The requirement for informed consent was waived because of the retrospective nature of the study. Clinicopathological data were collected, including age at surgery, sex, nodule size measured in fresh specimens, bilaterality, multifocality, and pathology at diagnosis (FNA, FS, and FP). All the patients involved are Chinese population.

### Preoperative FNA and intraoperative FS evaluation

FNA biopsy is used to obtain cells from nodules in the thyroid gland. Pathologists subsequently make a diagnosis based on the biopsy samples. The Bethesda category was applied for FNA diagnosis (Bethesda I for nondiagnostic/unsatisfactory, II for benign, III for atypia of undetermined significance/follicular lesion of undetermined significance, IV for follicular neoplasm/suspicious for follicular neoplasm, V for suspicious for malignancy, and VI for malignancy). At our hospital, we conduct FNA mostly for patients with a thyroid imaging reporting and data system (TI-RADS) 4a and above. Notably, nodules highly suspicious for adenoma were not recommended for FNA. After the nodules were surgically resected, the pathologists immediately made an intraoperative FS diagnosis of the specimens. The FS diagnosis was stratified into malignancy, suspicious for malignancy, benign, and indeterminate.

### Statistical analysis

Categorical variables were compared using the χ2 test. A Sankey diagram was generated to visualize the shifts in diagnosis between FNA and FS, as well as FS and FP. The accuracy, sensitivity, specificity, positive predictive value (PPV), and negative predictive value (NPV) were calculated using standard formulae. In addition, the net reclassification index (NRI) was used to evaluate whether one model led to a better reclassification of patients than another model, using previously described (15, 16). Statistical analyses were conducted using SAS (version 9.4; SAS Institute, Inc., Cary, NC, USA) or R (version 3.5.2). P < 0.05.

## Results

### Clinicopathologic characteristics of patients

Between January 2012 and December 2016, 12826 patients underwent thyroid surgery at the First Affiliated Hospital of the Zhejiang University School of Medicine. 3807 patients with thyroid nodules who underwent all three pathological diagnoses (FS, FNA, and FP) in our hospital were included in our analysis. The demographic and clinicopathological characteristics of the patients are summarized in **Table 1**. The cohort included 2870 (75.4%) women and 937 (24.6%) men. Patients aged < 55 years accounted for 77.5% of the study population. There were 829 (21.8%) patients whose FNA nodules had a diameter < 5 mm, 1747 (45.9%) had a diameter between 5 and 10 mm, and 1231 (32.3%) had a diameter ≥10 mm. Bilateral tumors were observed in 735 (20.7%) patients, and multifocal tumors were observed in 1071 (30.2%) patients with malignant pathology.

**Table 1:** Clinicopathologic characteristics of the 3807 patients who met inclusion criteria.

	Final pathology							
	Total (n=3807)		Malignancy (n=3544)		Benign (n=263)			
Variables	No.	%	No.	%	No.	%	P value	
**Age (years)**							<0.001
<55	2949	77.5	2790	78.7	159	60.5	
≥55	858	22.5	754	21.3	104	39.5	
**Sex**							0.013
Female	2870	75.4	2655	74.9	215	81.7	
Male	937	24.6	889	25.1	48	18.3	
**Diameter (mm)#**							<0.001
<5	829	21.8	812	22.9	17	6.5	
5-10	1747	45.9	1652	46.6	95	36.1	
≥10	1231	32.3	1080	30.5	151	57.4	
Bilaterality								
Yes	NA	NA	735	20.7	NA	NA	
No	NA	NA	2809	79.3	NA	NA	
multifocality								
Yes	NA	NA	1071	30.2	NA	NA	
No	NA	NA	2473	69.8	NA	NA	

#, The diameter of nodules which underwent FNA; N/A, Not Applicable

### Visualization of diagnosis shifts amongst FNA, FS and FP

**Figure 2:**
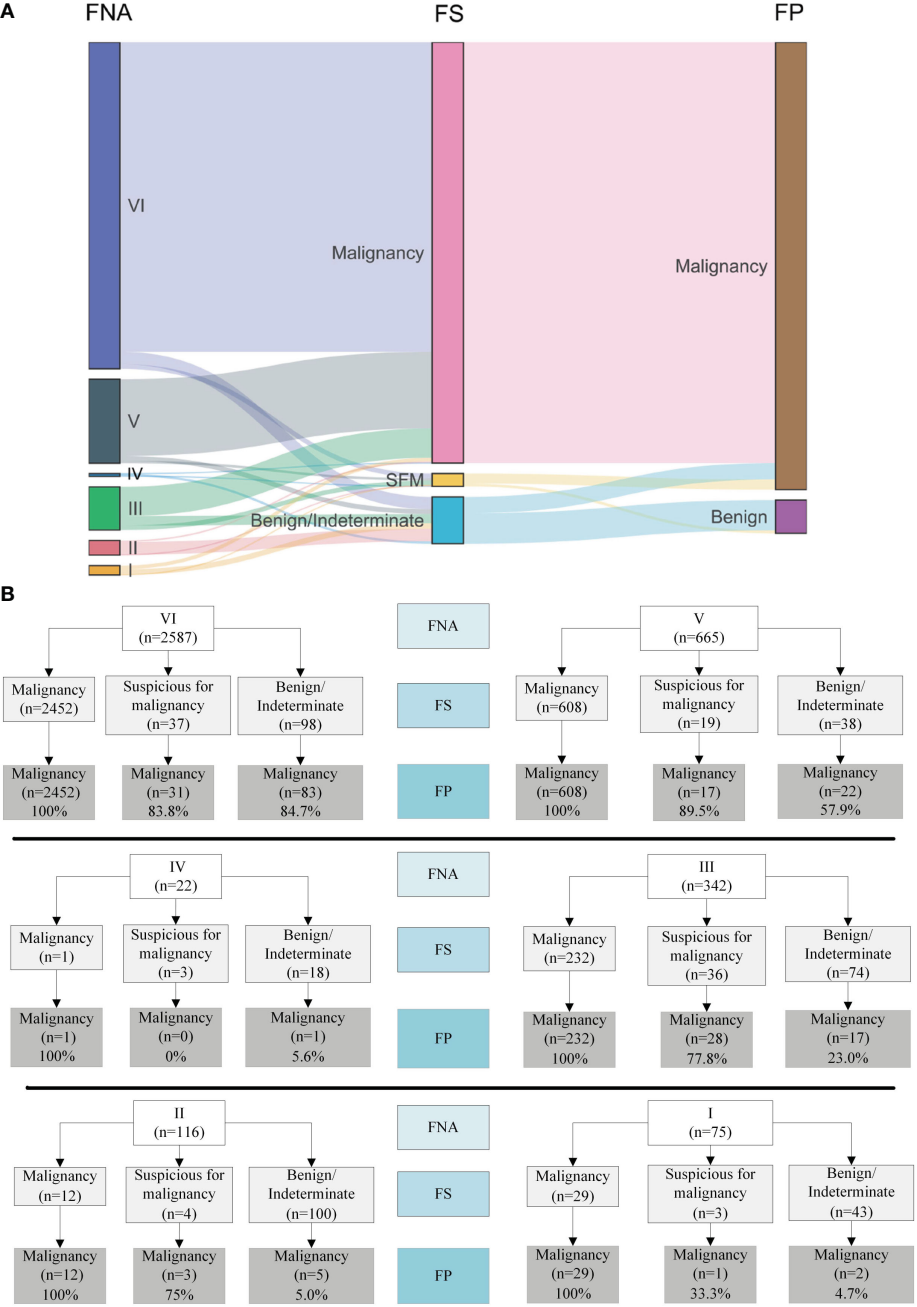
Visualization of the shifts of diagnosis among FNA, FS and FP. **(A)** Sankey diagram. **(B)** Details of the shifts. FNA I, II, III, IV, V, VI represents Bethesda category I (Nondiagnostic/unsatisfactory), II (Benign), III (atypia of undetermined significance/follicular lesion of undetermined significance), IV (follicular neoplasm/suspicious for follicular neoplasm), V (suspicious for malignancy) and VI (malignant).

A Sankey diagram was generated to visualize shifts in diagnosis among FNA, FS, and FP (**Figure 2A**). As shown in **Figure 2B**, most patients with Bethesda categories VI (2452/2587, 94.8%) and V (608/665, 91.4%) were classified as malignant when evaluated with FS. Notably, 67.8% (232/342) of the patients with Bethesda category III were also classified as having malignant nodules through FS. In addition, 9.7% (371/3807) of the patients were diagnosed with Benign or indeterminate FS histology (**Figure 2** and **Table 2**). Among the 371 patients, 98, 38, 18, 74, 100, and 43 patients were from Bethesda categories VI, V, IV, III, II, and I, respectively (**Figure 2B**).

**Table 2:** The distribution of final benign and malignant diagnoses among two diagnostic system.

Diagnosis Category	Final Pathology					
	Malignancy		Benign		Total	
	No.	%	No.	%	(N = 3807)	
FNA						
Malignant (VI)	2566	99.2	21	0.8	2587
SFM (V)	647	97.3	18	2.7	665
FN/SFN (IV)	2	9.1	20	90.9	22
AUS/FLUS (III)	277	81	65	19	342
Benign (II)	20	17.2	96	82.8	116
Nondiagnostic/unsatisfactory (I)	32	42.7	43	57.3	75
FS						
Malignancy	3334	100	0	0	3334
Suspicious for malignancy	80	78.4	22	21.6	102
Benign	126	34.5	239	65.5	365
Indeterminate	4	66.7	2	33.3	6

The malignancy rates were 99.2% (2566/2587), 97.3% (647/665), 9.1% (2/22), 81% (277/342), 17.2% (20/116), and 42.7% (32/75) for Bethesda categories VI, V, IV, III, II, and I, respectively (**Table 2**). Surprisingly, we found that all nodules that were malignant in FS were eventually diagnosed as malignant (100%, 3334/3334). Meanwhile, among the nodules classified as suspicious for malignancy, benign, and indeterminate through FS, the malignancy rates were 78.4% (80/102), 34.5% (126/365), and 66.7% (4/6), respectively.

### Diagnostic accuracy fine-needle aspiration, frozen section and final pathology

**Figure 3:**
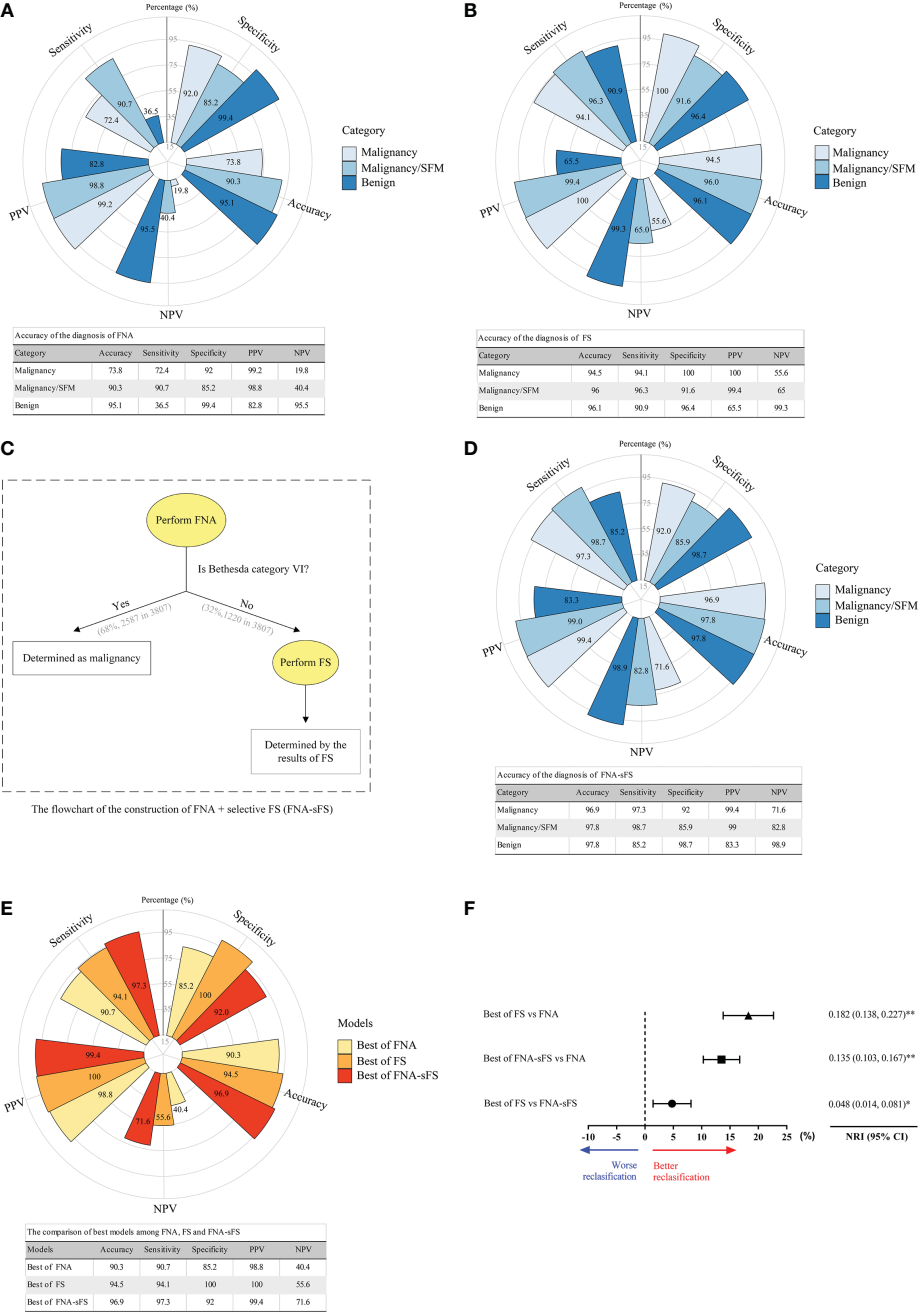
Accuracy comparison of the diagnosis of FNA, FS and modified diagnostic model (FNA+sFS). **(A)** Accuracy of the diagnosis of FNA. (All values shown as percent). **(B)** Accuracy of the diagnosis of FS. **(C)** The flowchart for FNA+ Selective FS (FNA+sFS). **(D)** Accuracy of the diagnosis using FNA+sFS. **(E, F)** The comparison of best models among FNA, FS and FNA+sFS. NRI, net reclassification index. * P < 0.05; ** P < 0.05. NPV, negative predictive value; PPV, positive predictive value.

To evaluate the ability of FNA and FS to distinguish between benign and malignant thyroid nodules, we compared the diagnosis of FNA and FS with that of the final pathological diagnosis (FP). When malignancy and nodules suspicious for malignancy were considered as the final malignancy diagnosis, the FNA diagnostic system achieved optimal discriminative power (accuracy, 90.3%; sensitivity, 90.7%; specificity, 85.2%; PPV, 98.8%; NPV, 40.4%) (**Figure 3A**). Similarly, the best model of the FS diagnostic system for distinguishing benign from malignant nodules had an accuracy of 94.5%, sensitivity of 94.1%, specificity of 100%, PPV of 100%, and NPV of 55.6% (**Figure 3B**).

To seek an alternative scheme, a flowchart of FNA+selective FS (FNA+sFS) was designed (**Figure 3C**), and only 32% of patients (1220/3807, Bethesda category I-V) needed to undergo FS for the construction of FNA+sFS. When reclassified according to FNA+sFS, the malignancy rates for malignancy, suspicion of malignancy, benign, and indeterminate were 99.4% (3448/3469), 75.4% (49/65), 16.7% (45/269), and 50% (2/4), respectively (**Table 3**). We found that the best FNA+sFS model had an accuracy of 96.9%, sensitivity of 97.3%, specificity of 92%, PPV of 99.4%, and NPV of 71.6% (all P < 0.05, **Figure 3D**). A comparison of the best models among FNA, FS, and FNA+sFS is shown in **Figure 3E**. Compared with the FNA diagnostic system, the FS correctly reclassified 18.2% of patients (NRI=0.182, 95% CI 0.138-0.227, P <0.001). The new proposed scheme, FNA+sFS, also performed much better than FNA alone (NRI=0.135, 95% CI 0.103-0.167, P <0.001, **Figure 3F**).

**Table 3:** The distribution of final benign and malignant diagnoses in new diagnostic system.

Diagnosis Category	Final Pathology				
	Malignancy		Benign		Total
	No.	%	No.	%	(N = 3807)
FNA+sFS					
Malignancy	3448	99.4	21	0.6	3469
Suspicious for malignancy	49	75.4	16	24.6	65
Benign	45	16.7	224	83.3	269
Indeterminate	2	50	2	50	4

### The validation of FNA+sFS in an independent cohort

**Figure 4:**
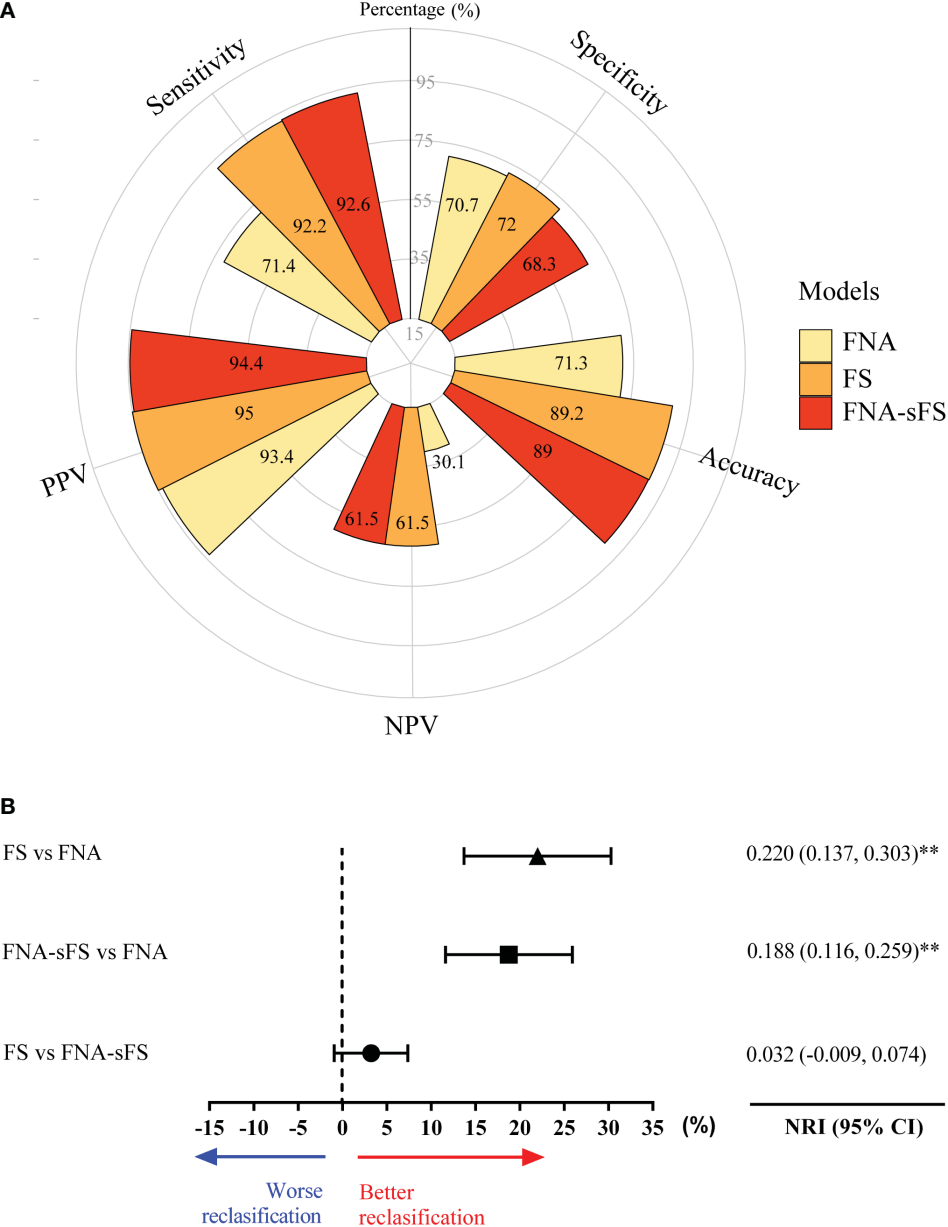
The validation of discriminatory capability of FNA+sFS in an independent cohort. **(A)** The comparison of best models among FNA, FS and FNA+sFS in an independent validation cohort. **(B)** The forest plot shows FNA-SF has better reclassification capacity than FNA and FS is also better than FNA, while there is no statistically difference between FNA-SF and FNA. NRI, net reclassification; ** P < 0.05. NPV, negative predictive value; PPV, positive predictive value.

Finally, an independent validation cohort of 554 patients from Hangzhou First People’s Hospital, Zhejiang Province, China (from May 2015 to March 2020) was included in this study and analyzed in accordance with the procedures described above (**Figure 4**). When FNA+sFS was applied to the independent validation set from the Hangzhou First People’s Hospital cohort (N=554), it displayed a discriminatory capability with an accuracy of 89%, sensitivity of 92.6%, specificity of 68.3%, PPV of 94.4%, and NPV of 61.5%, which was significantly better than FNA only (NRI=0.188, 95% CI 0.116-0.259, P <0.001) and not inferior to FS (P = 0.137).

## Discussion

Distinguishing malignant nodules from benign nodules plays an important role in thyroid disease management, especially in avoiding overtreatment and potential surgical complications. FNA and FS are well-established diagnostic procedures for malignancy prediction; however, their accuracy and necessity are still debated. We validated the clinical significance of FNA and FS in a large cohort of 3837 patients. We propose a combined FNA+sFS strategy to better balance the diagnostic accuracy and medical cost efficiency.

Diagnostic FNA has long been recommended as the first choice of treatment for nodules suspected to be malignant. In our cohort, it is notable that FNA alone can achieve 90.3% accuracy and 90.7% sensitivity when malignancy and suspicious for malignancy are classified as positive, which influenced the routine use of FNA as the first-line screening tool. However, we also observed that the specificity of FNA was only 85.2%. The undetermined cases may need a repeat aspiration or molecular tools to help decision-making (17). A previous study of a large sample size suggested repeat fine-needle aspiration of the atypia of undetermined significance/follicular lesion of undetermined significance (AUS/FLUS) nodules did not promote detection of malignancy (18). Additional molecular tools such as Afirma GSC and Thyroseq v3 only had probability of correct diagnosis of 63.7% and 73.2% with expenses of $17,873 and $14,277 per each correct case (19). Molecular tools are inaccessible in many countries including China. Additionally, patients may refuse repetitive aspiration for various reasons.

Intraoperative FS was another option for secondary confirmation. FSs are routinely used in China and other Asian countries (13, 20, 21). For patients with intermediate and nondiagnostic FNA cytopathology, FS might help to confirm the necessity of total thyroidectomy and central neck lymph node dissection. A retrospective study of 662 patients by Chang et al. demonstrated that FS could lead to a correct diagnosis in 78.9% of lesions, while FNA was only 21.1% (22). Sandrine Estebe et al. found that unnecessary total thyroidectomy and two-stage surgery rates would decrease from 3.6% and 7.7% to 0% and 5.2%, respectively with FS (23). On the other hand, Reema Mallick et al. explored the usefulness of FS and found that intraoperative management was changed in only 2.1% of their cohort and therefore did not recommend its routine use (24). In the present study, we validated the accuracy and necessity of FS by using a fairly large sample size of 3807 patients. FSs demonstrated an overall 18% extra reclassification better than FNA, which confirms the clinical value and necessity of FS, with all categories benefiting.

Our data showed a high malignancy rate of 99.7% in FNA category VI. A meta-analysis by Nguyen Truong Phan Xuan et al. also demonstrated an extremely high malignancy rate of 98.1% %Bethesda VI nodules in Asian countries (20). Therefore, it is reasonable not to apply FS to this subset of patients. Some previous studies also suggested eliminating the use of FS in high categories of FNA, which is in agreement with our strategy of leaving Bethesda VI nodules from the frozen section procedure (25, 26). For nodules with FNA categories I-V, we further investigated the necessity of performing FS with FNA and found that selective FS could raise the rate of correct reclassification by 15% in comparison to FNA only (accuracy 96.9%, sensitivity 97.3%, specificity 92%, PPV 99.4%, and NPV 71.6%). Besides, the cost of FNA was nearly 170 USD and the cost of FS was about 20 USD for each specimen. The potential benefits of the FNA+selective FS would decrease about 1/3 usage of FS while keeping high discriminative power with accuracy of 96.9%, sensitivity of 97.3%, specificity of 92%, PPV of 99.4% and NPV of 71.6%.

Our study had some limitations. First, we did not include molecular tools to increase the accuracy of the prediction. Supplementary molecular tools, such as Afirma GSC or Thyroseq v3, are not currently available in China. In addition, as a retrospective analysis, although we involved an analysis of an external independent cohort consisting of 554 cases from another large clinical center, there is still room for further analysis to approach the highest level of reliability. We recommend setting up a prospective multicenter randomized clinical trial (RCT) with detailed pathology subtypes and supplementary molecular information to optimize our “FNA + selective FS” system in the future.

In summary, our study retrospectively evaluated the clinical value of fine needle aspiration and intraoperative frozen sections in a large sample size of 3837 patients. We propose a modified thyroid nodule prediction method of “Combined FNA and selective FS,” which helps clarify the clinical criterion of FS usage with consideration of both diagnostic accuracy and medical efficiency.

## Data availability statement

The original contributions presented in the study are included in the article/supplementary material. Further inquiries can be directed to the corresponding authors.

## Ethics statement

The studies involving human participants were reviewed and approved by Institutional Review Board of the First Affiliated Hospital of Zhejiang University School of Medicine Institutional Review Board of Hangzhou First People’s Hospital. The patients/participants provided their written informed consent to participate in this study.

## Author contributions

ZM, YD, LW, WW and LT designed the current study and wrote the manuscript. ZM, YD and LW conducted the statistical analyses. ZM, YD, LW, YZ, GW, QY and JW created the original databases to collect the clinicopathological data. WW, DL and LT reviewed and revised the manuscript. All authors contributed to the article and approved the submitted version.
